# A Wearable Body Controlling Device for Application of Functional Electrical Stimulation

**DOI:** 10.3390/s18041251

**Published:** 2018-04-18

**Authors:** Nazita Taghavi, Greg R. Luecke, Nicholas D. Jeffery

**Affiliations:** 1 Department of Mechanical Engineering and Virtual Reality Applications Center, Iowa State University of Science and Technology, Ames, IA 50011, USA; 2 Department of Veterinary Medicine & Biological Science, Texas A&M University, College Station, TX 77843, USA; njeffery@cvm.tamu.edu

**Keywords:** functional electrical stimulation, spinal cord injuries, bionic test-bed, balancing device

## Abstract

In this research, we describe a new balancing device used to stabilize the rear quarters of a patient dog with spinal cord injuries. Our approach uses inertial measurement sensing and direct leg actuation to lay a foundation for eventual muscle control by means of direct functional electrical stimulation (FES). During this phase of development, we designed and built a mechanical test-bed to develop the control and stimulation algorithms before we use the device on our animal subjects. We designed the bionic test-bed to mimic the typical walking gait of a dog and use it to develop and test the functionality of the balancing device for stabilization of patient dogs with hindquarter paralysis. We present analysis for various muscle stimulation and balancing strategies, and our device can be used by veterinarians to tailor the stimulation strength and temporal distribution for any individual patient dog. We develop stabilizing muscle stimulation strategies using the robotic test-bed to enhance walking stability. We present experimental results using the bionic test-bed to demonstrate that the balancing device can provide an effective sensing strategy and deliver the required motion control commands for stabilizing an actual dog with a spinal cord injury.

## 1. Introduction

### 1.1. Background

Most quadrupeds develop their walking ability at an early age. During walking, the animal generates the forces necessary for movement using the flexor and extensor muscles. While the brain generates the signals required for balance and walking, the spinal cord plays a key role in transmission of signals and activation of the walking muscles that produce specific patterns for swing and stance. If the spinal cord is damaged, the animal may lose its ability to walk or maintain balance [1]. Spinal cord injuries are widely recognized as one of the most debilitating injuries in both animals and humans. Unfortunately, repairing the damage in spinal cord is an unsolved challenge for neuroscientists and regeneration of nerve cells is not possible in either humans or animals. Therefore, most patients who sustain spinal cord injuries suffer disabilities with no promising therapies.

Along with research by neuroscientists looking for treatments to repair the nerve damage and restore walking abilities, engineers are also working to restore walking activity by applying both engineering techniques and biological knowledge to devise new and innovative mechanical devices that correct the disability. We categorize these devices into two types: “supportive” which are designed to reinforce the body and enhance remaining abilities, and “neural stimulation” to repair or replace the damaged neural systems.

Perhaps the simplest of the supportive devices are orthoses that are worn by those patients with limited walking abilities to provide an alternate path of mechanical support to the affected limbs during standing and walking [2,3]. These devices do not use any actuators to move or control the limbs. However, powered exoskeletons are available, such as the Argo Medical ReWalk (ReWalk Robotics Ltd., Yokneam, Israel) and HAL-5 (Tsukuba University and Cyberdyne, Tsukuba, Japan) which use DC motors to move limbs and control joints when they are worn by the patient [4,5,6]. Some of these exoskeletons are also designed to provide the ability to carry larger load capacities for even a healthy user, such as the Berkeley lower extremity exoskeleton (Berkeley, University of California, Berkeley, CA, USA) [7,8].

Although exoskeletons and orthoses are very promising for achieving walking restoration, there are also some disadvantages [9]. The limitations include the strength and weight of the devices, battery capacity, actuator noises, and material costs. An additional problem is that these devices must be custom-designed for each individual.

Neural engineering devices are being developed that use the FES technique in an effort to overcome the shortcomings of exoskeletons and orthoses. These FES devices are designed to control nervous system disorders by applying electrical stimulation directly to the nerves. The FES approach uses a small electrical current applied to the nerve innervating the muscle using electrodes on the skin [10], or using electrodes implanted directly to the stimulating nerves. The aim is to improve or stimulate remaining biological functions after injury. One type of neural prostheses can deliver FES directly [11]. These devices usually consist of electrodes directly attached to the nervous system. Charge injection through these electrodes into the tissue can restore some of the abilities lost by injury [12,13,14,15]. There are two kinds of electrodes in this case: surface electrodes and implanted electrodes.

Surface electrodes may be placed on the skin to activate the underlying tissue [16,17] while implanted electrodes are placed directly in contact with targeted tissue by surgery [18,19]. Surface electrodes are safer and can be applied quickly on the body. However, they need larger current to excite the targeted tissue. Implantable microelectrodes can excite even deep muscles by applying small currents, but the system design is complicated due to the need for surgical implantation and precise application of low-level current. In addition, these microelectrodes must be durable and resistant to corrosion and mechanical failure in the body.

Intraspinal microstimulation (ISMS) is a new application of FES that uses microwires implanted into the spinal cord to excite neural networks. Wires are placed in a small region and require less current than some other FES techniques to produce the movement [20]. Results of some previous studies indicate that this technique can be a fatigue-resistant method to produce movement [21]. The feasibility of this method has been tested on cats and results show great promise [22].

As an example of neuroprostheses, some research has focused on developing human-made electronic dura mater (e-dura), which has been fabricated and tested on rats for long-term neural interfaces [23]. E-dura consists of a substrate, interconnects, soft electrodes and a fluidic microchannel. The implant has the shape and mechanical properties close to the host dura mater tissue to provide functionality and long-term bio integration within the spinal cord. Electrodes and interconnects of e-dura can transmit electrical charges while the microchannel transfers drugs. The design of this device also provides capability below the e-dura for the placement of other implants.

Neural prostheses with skin electrodes have been developed for various intentions such as exercise, physiotherapy and reduce pain and spasticity. Neural retraining is another clinically acceptable reason for using some neural prostheses with attachable electrodes on skin. Portable FES devices for stimulation of nerves with skin electrodes to counteract footdrop in hemiplegic patients are an example of these sort of neural prostheses [24,25,26,27]. Lindquis et al. [28] studied the application of FES on a group of tibialis muscles and its effects on counteracting footdrop and patients gait improvements during treadmill gait training using an open loop feedback control. Their portable FES device includes a sensory insole inside the shoe to sense the touching time of the patient’s foot heel on the floor. The stimulator, receiving data from the sensor, activates target muscles during swinging gait phase through a pair of skin electrodes. These studies proved that the electrical stimulation can assist patients for nerve retraining and improve gait during treadmill gait training. Cho et al. [29] used the similar device to investigate effects of electrical stimulation applied to both tibialis anterior and medius muscles on patients trained on moving treadmill. The results of their experiments also showed patients received stimulations on both groups of muscles improved their gait during four weeks of practice more than patients exercising without application of FES on their muscles. Seel et al. [30,31] proposed and showed the effectiveness of an automatic feedback control to simultaneously control foot pitch and roll motion in drop foot patients using iterative learning control.

Detecting gait events is another emerging subject in the field of gait control and the correction of walking abnormalities. Pressure, inertial and magnetic sensors have been used widely to detect gait phases and events. Chia et al. [32] developed a biomimetic multi-channel neural prosthesis and an adaptive algorithm to accurately detect six gait sub-phases in real time. Their device uses two wearable inertial and/or magnetic sensors attached laterally on the shanks [33]. Foglyano et al. [34] implanted a multichannel system with intramuscular electrodes on the nerves for muscle stimulations. An external rechargeable control unit controlled the stimulation parameters and produced power. A separate wireless inertial measurement unit was used to initiate steps, determine gait intentions, and control stimulations.

Electrodes or wires are used in any FES method, and the charge injected through electrodes or wires must be controlled to produce desired movement, such as a desired walking gait. To restore walking, skeletal muscles of the limbs must be activated in a coordinated manner. These muscles, in turn, produce the required forces for the movement of bones. Many canine patients can move their limbs reflexively in a fairly effective walk, but they are not able to balance their body or walk with a normal gait pattern. Our goal is to develop the required electrical excitation so that the target muscles will stabilize the body during walking when normal neural control of the limbs is damaged and thus restore balance and walking.

Our current work concentrates on developing a device to restore walking stability in dogs with spinal cord damage, specifically focused on Dachshunds. Dachshunds are good subjects for walking restoration because these chondrodystrophic dogs are highly susceptible to spinal injuries due to an accelerated rate of intervertebral disc degeneration. We develop our approach to restore ambulation of small dogs as a first step to pave the way to new engineering methods for restoring walking in humans.

**Figure 1:**
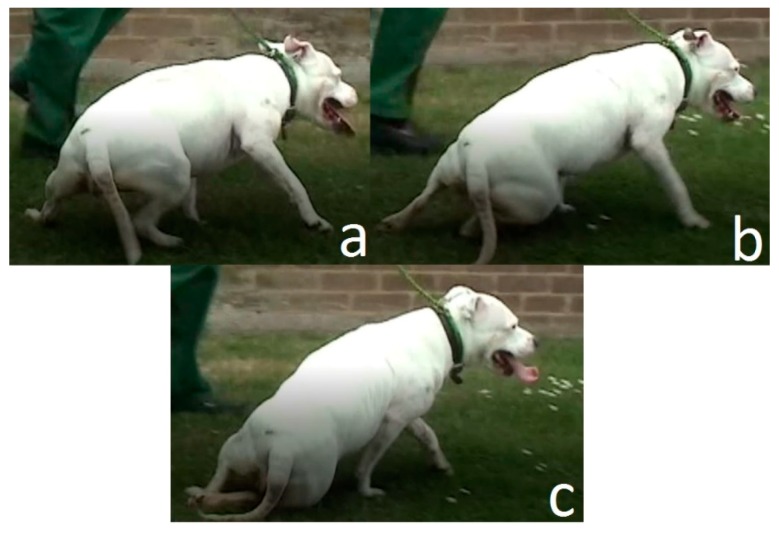
(**a**) Injured dog at balance, (**b**) Dog at the critical timing before falling, (**c**) Dog has fallen.

In dogs with damaged spinal cords, the neural communication between the brain and the rear legs are lost. Many injured dogs retain a limited ability to walk, because reflex signals continue to stimulate the movement of the hind legs when the feet touch the ground. However, without full neural connections between the hind legs and the brain, the dog loses full body control of the rear legs and cannot maintain balance. Usually, during walking, the hindquarters are tilted toward one side before falling as shown in Figure 1. 

We have focused on the development of a device that can be attached to the pelvis of the dog to measure the angular of rotation of pelvis. Any abnormally large rotation angle of the pelvis can be a signal to an impending fall and can be used to generate corrective signals to the hind legs.

Prior to testing the device on a real dog, we have designed and built a bionic robot canine to model the pelvis and hind limbs of Dachshunds. Several different robots have been developed with designs inspired from animals and quadrupeds [35]. Morrey et al. [36] developed a small robot with the name of “mini-whegs” to jump and run. Their design was inspired from cockroach locomotion principles and it could be used for rescue applications. Another example is ATRIAS [37], which is a three-dimensional bipedal with light legs, six actuators 13 degrees of freedom to produce walking gait. Maybe one of the most famous robots in this field is “BigDog” [38], built by Boston Dynamics (Boston Dynamics, Waltham, MA, USA). This is a rough-terrain robot that can walk with natural speeds, balance and carry loads in places inaccessible to wheeled vehicles. Our robot is unique because we use it for a test bed for algorithm development. We use the bionic test-bed to explore the sensor response to various gait conditions, the effects of various assumptions about the restricted degrees of freedom of the dog and rear legs, to develop and validate the control algorithms for achieving balance and stability, and to gain insight into the relative magnitude of the stabilizing corrective signal necessary.

In this work, we first study the walking gait of a normal dog. We simplify the kinematic motion of the actual dog hip, knee and hock, and our experimental results show that we produce a relatively normal walking gait, even while assuming little or no motion of the knee joint. We develop the kinematics of our bionic robot to allow us to mimic the stance and gait of the normal dog. We test the functionality of the balancing device and develop balancing approaches on our bionic dog test-bed. This knowledge provides a basic walking gait on the robotic test bed, and we will add small corrective signals to help provide balance as the dog start to fall sideways. We will develop and test stabilization algorithms on our robotic test bed in both standing and walking modes. Experimental results substantiate the operation of our test-bed, the walking gait of the dog, and the performance of our initial balancing algorithm 

In previous work [39], an earlier version of the test-bed was developed with a single motorized axis, based of veterinarian experience that the hock joint was used by dogs as the primary balancing joint. This work demonstrated the efficacy of using the bionic test bed for development, but also pointed to the need for a more complete hindquarters model of the dog. Our current work stimulates both hip and hock joints to affect the stabilization strategy.

### 1.2. Motivation and Novelty

Designing a test bed for evaluating a special neural injury and conducting tests using a rehabilitation model based on FES is a challenge for both neuroscience researchers and engineers. In some cases, in order to model an abnormal gait event and test the effect of proposed corrective model, an animal is intentionally mutilated. Sometimes, the injury to the animal is permanent. An example is the work of Park et al. [40] where the objective was to model an uneven gait to test the effects of electrical stimulation for gait correction. This test-bed included two adjacent treadmills capable of operating at different speeds. Each treadmill was equipped with specialized pressure sensors to record the foot-to-ground contact time and position in order to establish the step length. Cuff electrodes were used to apply the augmentation stimulus that was based on feedback from the pressure sensors under the treadmills. The animal was sedated to calibrate the magnitude of the required stimulus, and the objective of the experiment was to change the gait of the animal while walking. These sorts of experiments demonstrate the effectiveness of FES for gait correction, but introduce other challenges for the researchers, including specially designed augmentation algorithms, unrealistic sensor requirements, and the possibility of injury to a healthy animal for sedation during setup and testing. Moreover, every disabled animal is unique and suffers specialized injuries. In this work, we use inexpensive sensors, which will be attached to the animal later for feedback, and develop a generalized stabilizing algorithm based on feedback from these generalized sensors.

We develop and use this robotic test-bed to completely remove the need for a real animal during our initial experiments, and to provide a system for tailoring the FES inputs to a particular animal using the mechanical testbed. The robot uses well recognized control and robotic principles, which makes it inexpensive, easily programmable, kinematically accurate, and appropriate for use by nontechnical personnel.

The microcontrollers running this robot are two Arduino Uno boards (Arduino and Adafruit Industries, New York, NY, USA), one dedicated to the stability augmentation sensing and control. While the robot is easy to program, it can easily provide the required motion of a real animal. The process for converting data from VICON software (VICON Motion Systems, Oxford, UK) to the gait trajectory is shown, based on Fourier approximation. We model a specific normal gait as an example, but our process can also be applied to an abnormal gait. While our experimental results show the performance of the stabilization algorithm in response to perturbations of the normal gait, our algorithm is based on sensor data showing the attitude of the animal, rather than on any specific gait. This provides the researcher with a tool that can be tailored to mimic the specific patient animal, beginning with gait development using the VICON and Fourier approach, validating the magnitude and performance of the stabilizing augmentation signal, and only then attaching the sensors and stimulation hardware to the animal.

## 2. Materials and Methods

### 2.1. Mechanical Model of Replica

**Figure 2:**
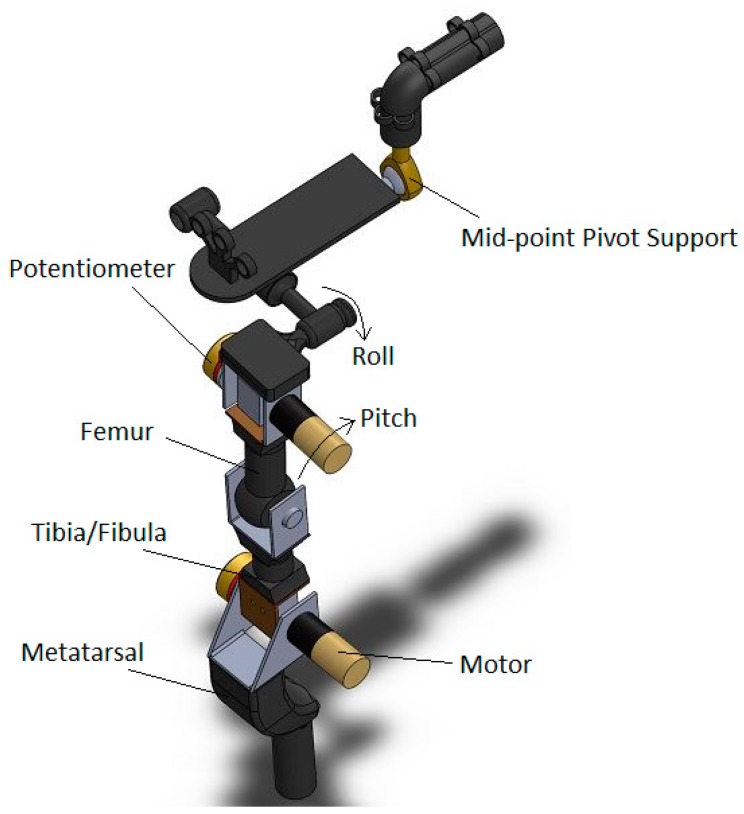
3D model of replica.

We designed the robot test bed to be physically representative of the adult dachshund hindquarters. The two legs of the front quarters are forward of the damaged spinal cord, and are still under the direct control of the dog. We assume that the front limbs provide support and stability for the forward half of the body, and replace these with a fixed mount for the mid-body of the dog that allows for appropriate rotation while also providing a measure of stability from the front quarters. The rear end of the robot includes the pelvis and two rear legs. Each leg includes 3-D printed plastic parts, which play the role of femur, tibia/fibula and metatarsal bones in the actual dog. The femur is attached to pelvis by the hip joint. The hip joint is designed to provide two rotational degrees of freedom in pitch and roll. Only the pitch rotation of this joint is controlled by a motor, and is used to mimic the change in hip joint angle during walking. The roll rotation allows the dog to move the leg laterally in and out from the centerline of the body. We have added a compliant spring to constrain the roll motion, based on veterinarian experience that ligaments and other connective tissue perform a similar function. The distal end of the femur is fixed to the tibia at the knee joint. In subsequent sections, we will show that kinematic evaluation of the normal dog gait indicates very little motion of the knee joint, and so we have simplified our bionic dog leg with a fixed knee joint. The hock joint, linking tibia and metatarsal, is also designed with motorized actuation. We provide rotation and control to match the motion found in this joint on the typical dog. The model of one leg and pelvis of our robotic dog test-bed is shown in Figure 2. The length of femur, tibia/fibula, and metatarsal and toes are 70, 70 and 105 mm, respectively. These bone sizes are based on x-ray image of the typical Dachshund dog [35].

The hip and hock rotational joints include a motor, gearbox, and rotational potentiometer. The rotational position of each motorized joint is controlled using an Arduino Uno microcontroller and Arduino A000079 Motor Shield, R3 (Arduino and Adafruit Industries, New York, NY, USA) [41]. The potentiometers measure the joint angles and provide position feedback to Arduino for control and stabilization. We use two Arduino boards to control the four joints of two robot legs. The two Arduino boards are connected by means of serial communication using pins TX/RX for communication and simultaneous operation. 

Analysis of walking motion in injured dogs shows that there is a critical pelvis position during an abnormal gait that is a precursor of the animal falling. Our aim is to identify this critical timing and immediately inject an electrical charge to actuate the proper muscles as to prevent the fall and continue walking. To this end, our approach is to first develop a normal walking gait on our robotic test-bed. Then we introduce the disturbances that induce falling of the hind quarters. We do this initially when the test-bed is stationary. We next develop a device with the proper sensors and a stabilizing control algorithm that will recognize the critical orientation and deliver an augmented electrical command to balance the dog. In the actual animal, the electrical charge to prevent falling and restore a normal gait will be delivered using either implanted or surface electrodes.

The injured animal retains existing residual walking muscle stimulation, and we design our device to augment the muscle function, rather than completely replace and control the muscle stimulation. The sensors and computational hardware on our “balancing device” must be light and portable, since it will be attached on body of the patient. We have selected the Arduino series microcontroller as a sensor interface and for control signal generation, and we use the Pololu #2470 [42], combination 3-axis accelerometer, 3-axis rate gyro, and 3-axis magnetometer as our Inertial Measurement Unit (IMU) sensor. The combination of this microcontroller and IMU make a small and light device that can be carried easily by patient.

**Figure 3:**
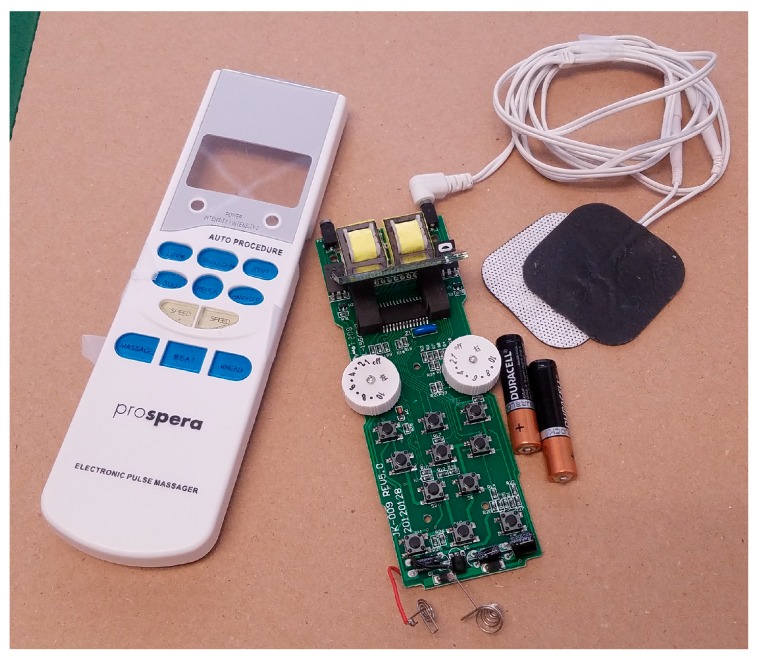
Small scale muscle stimulator used for preliminary tests.

The balancing device must also be able to deliver a precise electrical charge to electrodes on the muscles. We program the Arduino to process measurement data from the IMU and apply the commands needed to modify the muscle motion during walking. On our test-bed, the commands are computed and added to the motor gait commands. On the patient dog, these commands will be injected in order to stimulate the balancing muscle motion, either through surface electrodes or surgically implanted electrodes. Although the microcontroller cannot generate the high voltages necessary to generate the required stimulation current, we have done limited testing using the Arduino as the trigger for pulses using commercial hand-held massage machines, as shown in Figure 3. Although the electronics on these devices are small and light, the corrective currents required are also small enough that these types of devices may be sufficient for our applications.

**Figure 4:**
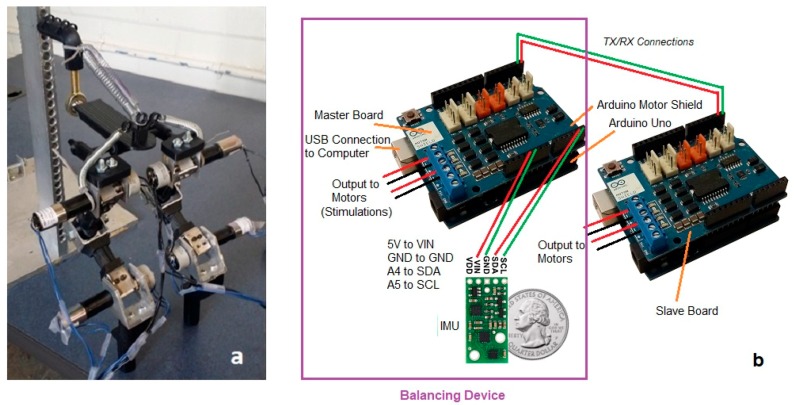
(**a**) The replica, (**b**) Arduino boards, IMU, and electronic connections.

We use our IMU attached to the pelvis to sense the horizontal angle of the pelvis. For our test-bed, we designate one of the Arduino controllers as the master, in charge of reading the IMU for balancing control, as well as controlling the hip and hock joint of the right rear leg using the feedback potentiometers and the joint motors. The second Arduino is designated as the slave controller, and is used only to control the motion of the hip and hock joints of the left rear leg, so is not part of the balancing device. The actual hardware for the bionic test-bed is shown in Figure 4. We start by analyzing the walking gait of normal dogs to identify the motion and orientation of the body during normal activity. As part of the balancing device, we use our IMU attached to animal hindquarters to measure speeds and accelerations. We use the IMU to determine the angle that the sensor makes with the ground. The sensor is mounted on the pelvis of the robotic dog, and would be worn on the back of the patient dog. The accelerometer and rate gyros in the IMU are combined to provide a measurement of the angle of the pelvis with the ground. Essentially, the gravity vector is measured by combining the three accelerometer readings so that we know which way is “down” relative to the dog pelvis, although we also combine the gyroscope information to help filter the noise using “Direction Cosine Matrix filter” [43]. This IMU measurement of the pelvis angle allows us to identify the critical timing during walking that will indicate an impending fall.

We continuously monitor the rotation angle, or roll angle, using the IMU, and as the angle gets too large, the dog is at risk of falling. While the critical value of this roll angle depends on the individual dog, we used video of injured dogs along with tests run on our bionic dog to determine an approximate value to use in our testing. We selected a threshold of 10–15 degrees as our critical value for hip rotation. To measure roll angle of dog pelvis during walking, the IMU is placed on the pelvis of the dog and it is connected to the master Arduino board. When used on the real dog, this Arduino will not only read the roll angle from the IMU, but it will also be used to apply electrical charge to the target dog muscles to initiate balancing. The best muscles to receive electrical charge are flexor or extensor muscles rotating hip and hock joints.

### 2.2. Mathematical Simulation for Dog Walking Gait

The trajectory of the normal walking gait for typical dog have been previously studied [39] in order to identify the hip, knee and hock angles during walking. VICON [44] video tracking software was used to record the motion of markers attached to the hock, knee, and hip joint of an adult dog while walking normally on a treadmill at a speed of 5 km/h. Although the walking video we analyzed was not from a Dachshund, the leg motion is generally the same for all breeds. The walking speed we used is typical for large dogs; however, it is too fast for the smaller dog, and we scale the speed to one-third to allow us to study the walking gait of Dachshund.

**Figure 5:**
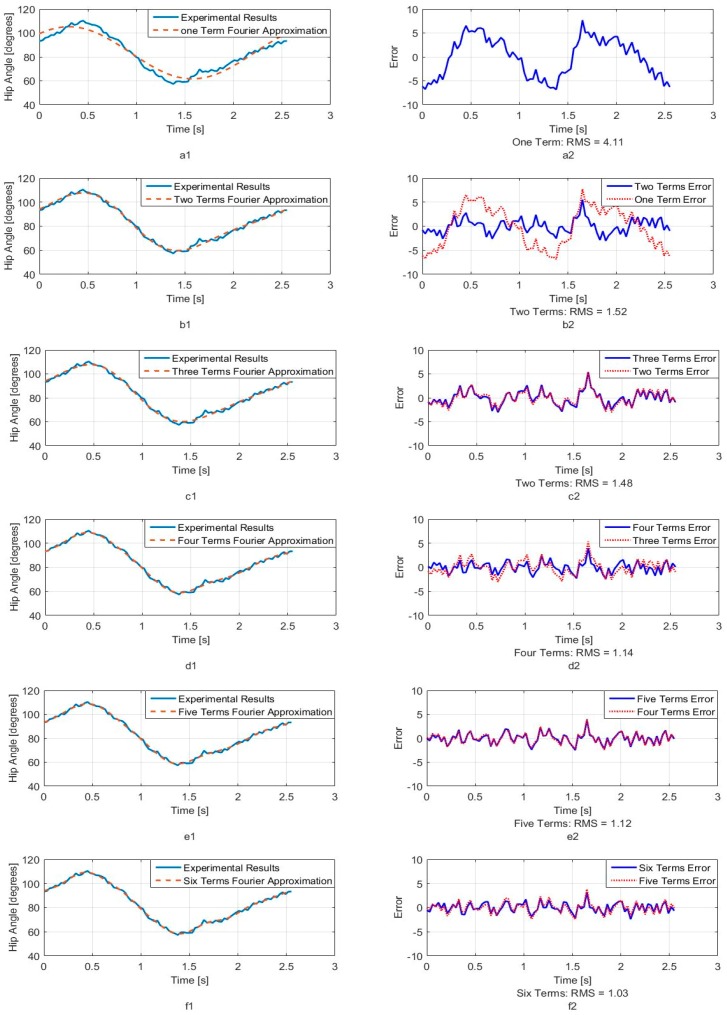
Hip joint angle as a function of time (**a1**) One term of Fourier series approximation (**a2**) One term approximation error (**b1**) Two terms of Fourier series approximation (**b2**) Two terms approximation error (**c1**) Three terms of Fourier series approximation (**c2**) Three terms approximation error (**d1**) Four terms of Fourier series approximation (**d2**) Four terms approximation error (**e1**) Five terms of Fourier series approximation (**e2**) Five terms approximation error (**f1**) Six terms of Fourier series approximation (**f2**) Six terms approximation error.

**Figure 6:**
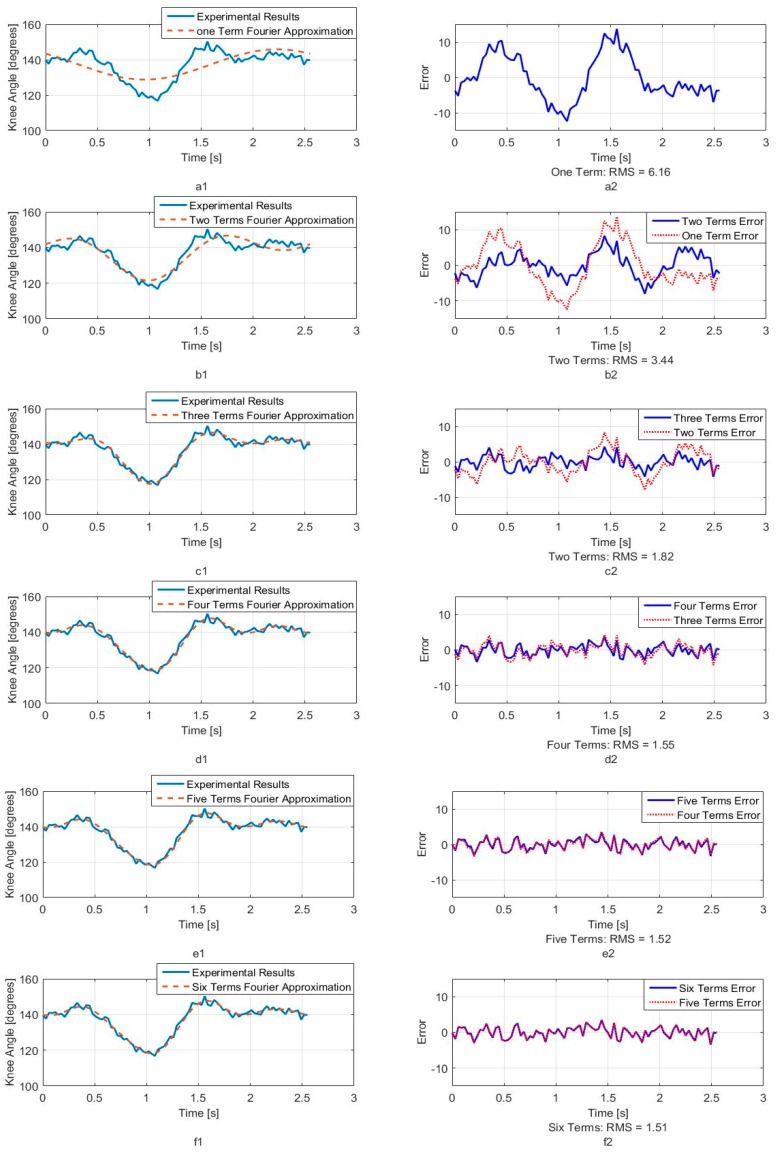
Knee joint angle as a function of time. (**a1**) One term of Fourier series approximation (**a2**) One term approximation error (**b1**) Two terms of Fourier series approximation (**b2**) Two terms approximation error (**c1**) Three terms of Fourier series approximation (**c2**) Three terms approximation error (**d1**) Four terms of Fourier series approximation (**d2**) Four terms approximation error (**e1**) Five terms of Fourier series approximation (**e2**) Five terms approximation error (**f1**) Six terms of Fourier series approximation (**f2**) Six terms approximation error.

**Figure 7:**
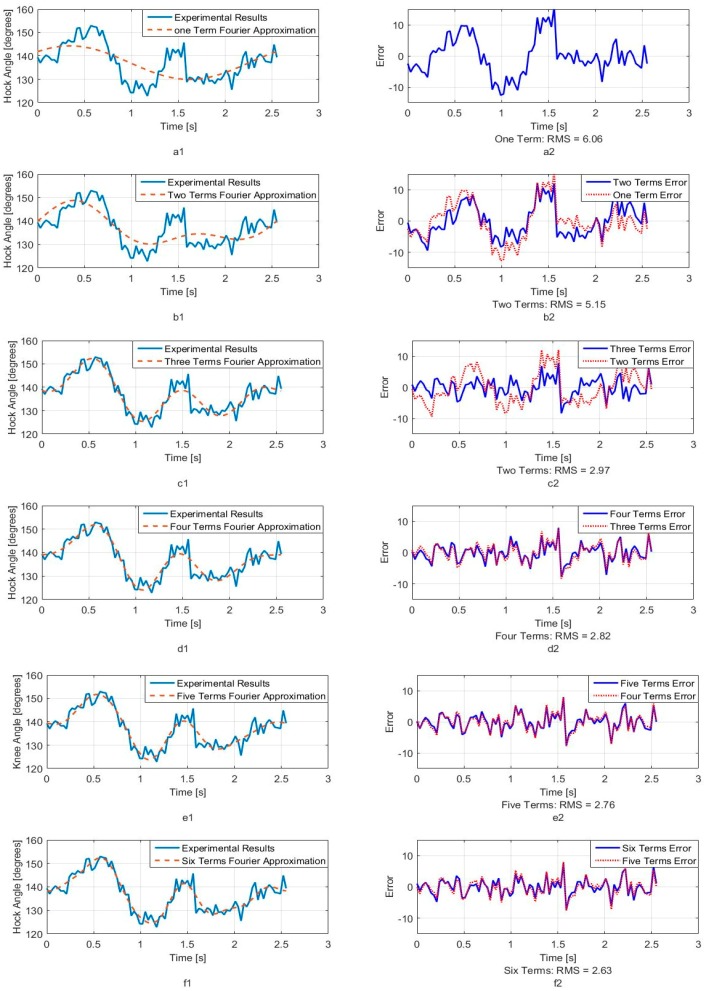
Hock joint angle as a function of time. (**a1**) One term of Fourier series approximation (**a2**) One term approximation error (**b1**) Two terms of Fourier series approximation (**b2**) Two terms approximation error (**c1**) Three terms of Fourier series approximation (**c2**) Three terms approximation error (**d1**) Four terms of Fourier series approximation (**d2**) Four terms approximation error (**e1**) Five terms of Fourier series approximation (**e2**) Five terms approximation error (**f1**) Six terms of Fourier series approximation (**f2**) Six terms approximation error.

Figure 5, Figure 6 and Figure 7 show calculated joint angles from the VICON video for hip, knee, and hock joints as discrete data points during time of one walking step. For each joint, the motion is cyclic from one step to the next. To represent the joint angles as continuous functions of time, we use the Fourier series of each time response as an approximation of the joint motion as follows:

(1) $$\theta_{\left(t\right)}=a_{0}+a_{1}\cos\left(\omega t\right)+b_{1}\sin\left(\omega t\right)+\ldots +a_{n}\cos\left(n\omega t\right)+b_{n}\sin\left(n\omega t\right)+\ldots$$

Here, $\theta_{\left(t\right)}$, is the angle in degrees, t is the time, ω is the fundamental Fourier frequency in rad/s and $a_{0}$, …, $b_{n}$ are Fourier constants used in the motion approximation. As our first approximation, we used six terms of Fourier series and calculated Fourier constants. These coefficients are shown in Table 1 with the fundamental frequency for a single step at *w* = 2.468 rad/s.

**Figure 8:**
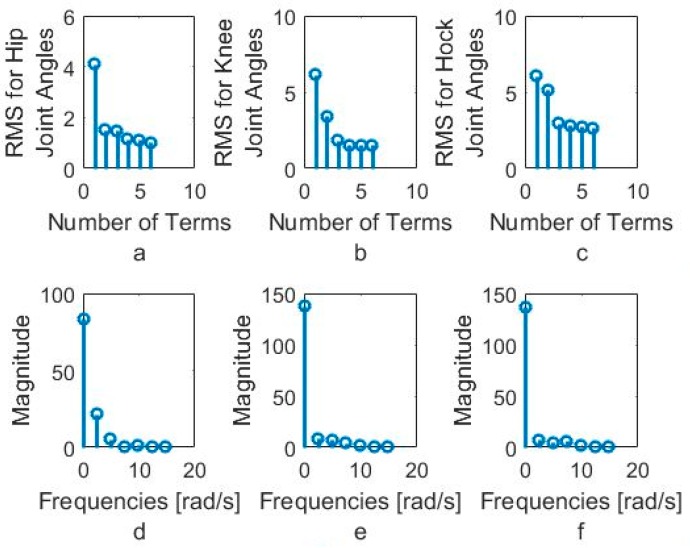
(**a**) RMS for hip joint angle, (**b**) RMS for knee joint angles, (**c**) RMS for hock joint angles based on the number of Fourier series, (**d**) Magnitude of Fourier series for hip joint, (**e**) Magnitude of Fourier series for Knee joint, (**f**) Magnitude of Fourier series for hock joint.

In order to better understand the number of Fourier terms needed to have the proper walking gait approximation, we have approximated each joint angle with just first Fourier term and calculated the error between approximated function and VICON experimental results as well as RMS error. Then we increased Fourier terms up to six Fourier terms and compared errors and RMS errors at each step. These results are shown along with the error for hip, knee and hock joint angles in Figure 5, Figure 6 and Figure 7 respectively. The RMS errors is shown as a function of the number of Fourier series for hip, knee and hock joints in Figure 8a–c. Figure 5 and Figure 8a show that just two Fourier series can give us good approximation for hip joint. Similar results are shown for knee in Figure 6 and Figure 8b and for the hock joint in Figure 7 and Figure 8c. We can conclude that three terms of Fourier series are enough for appropriate approximation for knee and hock joint angles. If we increase the number of terms, we obtain only slightly more accurate functions.

Figure 8d–f show the magnitude of the steady-state and subsequent terms in the Fourier series at each frequency for hip, knee and hock joint angles functions. These magnitudes confirm our findings using the RMS error to determine the number of Fourier terms to use. From Figure 8d, for the hip angle, we see the magnitude of the third and subsequent terms get very small indicting we can use just the first two terms for an accurate approximation. For the knee and hock joints, Figure 8e,f show that we can use just first three terms for an accurate approximation of the joint trajectories.

Later, we use six terms of Fourier series for all joints to mimic the gait of the dog with our bionic test-bed and with highest possible accuracy that our system can provide although we have proved the three terms are appropriate enough for mimicking dog walking gait.

**Figure 9:**
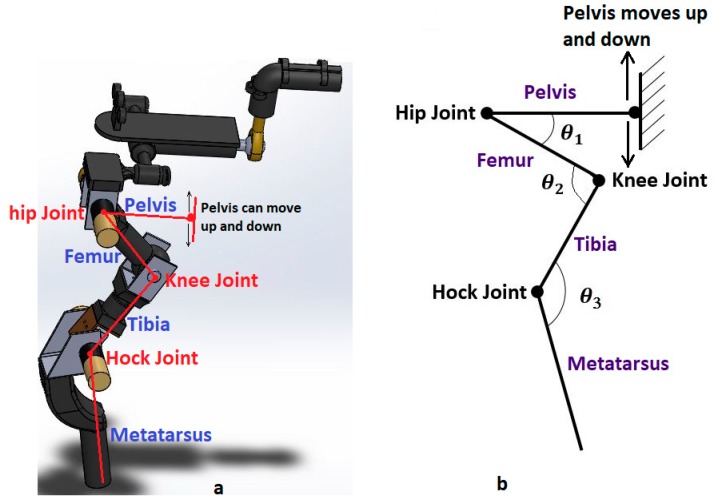
Three-link manipulator model. (**a**) Robot model, (**b**) Manipulator model

**Figure 10:**
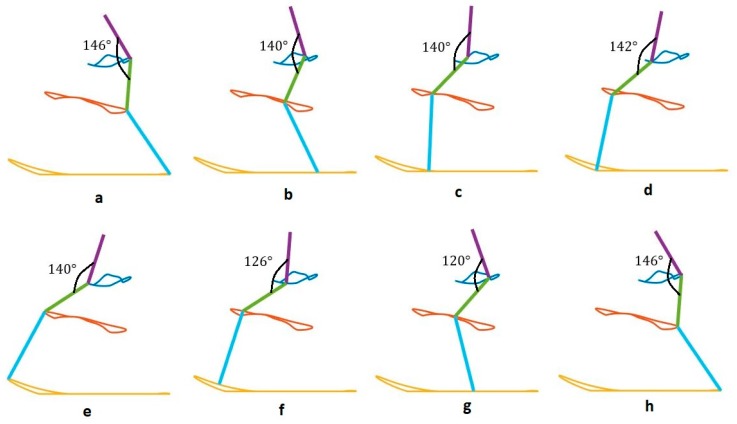
Bionic dog gait.

We use our bionic test bed to evaluate and simulate the movement of the actual dog. Each hind leg of the dog is modeled as a three-link open kinematic chain, as shown in Figure 9. The motion of the hip, knee and hock joints, $\theta_{1}$, $\theta_{2}$ and $\theta_{3}$, are generated using the Fourier time functions and can be used to simulate normal walking of the healthy dog using our three-link model. The leg motion trajectory for one complete step is shown in the snapshot sequence in Figure 10. This sequence is based on the Fourier approximation from Table 1. The stance phase of the leg, where the foot is in contact with the ground and the dog is moving forward, is shown in the top row, and we have highlighted the knee joint angle as the dog takes a forward step. It is clear that there is very little motion of the knee joint during stance phase. This corresponds to the time in Figure 6 before 0.6 s and after 1.8 s. The bottom row of four pictures shows the leg swing to reposition the foot for the beginning of the next step. This time sequence is shown in Figure 6 between 0.6 and 1.8 s. In the normal dog, as the leg swings forward, the foot and toes do not touch the ground, although they may come close or even lightly scrape. Because the dog has full feeling and control, there is a complex combination of hip, knee, and ankle motion during the swing forward such that we keep the foot off the ground. However, none of this motion imparts any forces between the ground and the hip. In order to balance the injured dog, we activate the stimulation on the leg muscles at stance phase. For our bionic dog, we can simply choose our own hip, knee, and ankle trajectories for the swing forward phase such that we keep the foot off the ground, and we choose to keep the knee joint fixed at the constant angle found in the stand phase which is 140 degrees. This means that the knee joint stays at a constant angle and does not require actuation.

The fore and aft movement of the hip is constrained on an actual dog by connection with the front legs, and on the test-bed, this constraint is provided by the fixed riser, which simulates the connection to the forward half of the dog. The ball and socket joint at the hip still allows the rotational movement between the front and rear parts of the torso. When robot dog toes touch the ground, they can push the hip upward. The ground constrains the foot and toes to move along a flat trajectory. We use the experimental Fourier trajectory during the stance phase, as the leg moves aft relative to the dog, but we choose a slightly modified trajectory for the hip and ankle joints during the swing forward phase. The modified trajectory only changes the swing forward, when there is no contact between the leg and the ground. Thus, there is no change in the forces applied by the leg during the full gait.

**Figure 11:**
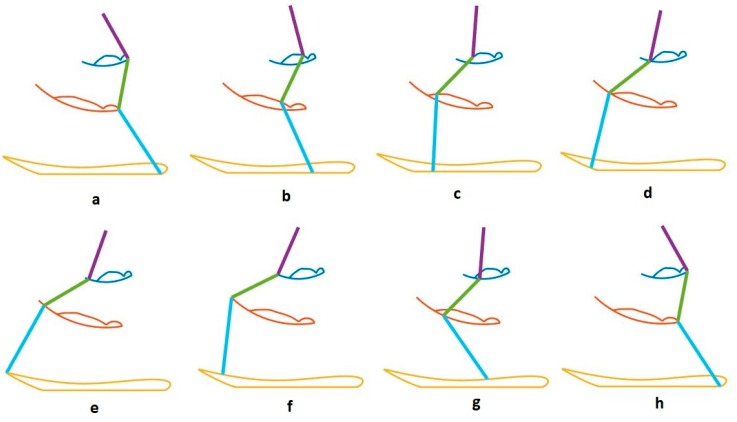
Bionic dog modified gait.

We show snapshots of the modified gait of bionic dog walking on the treadmill in Figure 11. The hip and hock joint angles are the same in both Figure 10 and Figure 11a–d and we show that the knee joint is almost fixed during this stage of walking. The joint trajectories are different between Figure 10 and Figure 11e–h. The differences are minor, and only occur during the swing forward phase when there is no contact between the foot and the ground, and so these differences do not affect the motion and balance of the bionic dog hindquarters. While the gait we produce for our test-bed is not an exact dog gait, it is still a good approximation for understanding the additional balancing stimulation necessary for correcting an abnormal gait and restoring stability and balance.

**Figure 12:**
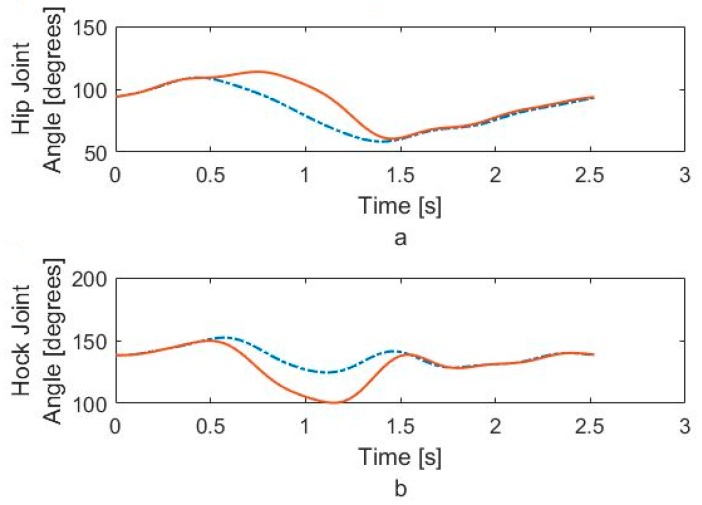
(**a**) Modified hip joint angle, (**b**) Modified hock joint angle.

To produce our simulated dog gait, we have modified both hip and hock angles in order that the robot dog lift its toes during walking. These modified angles for hip and hock joints, that can correct the gait of dog are shown in Figure 12a,b. The trajectory and joint angles match the experimental data during the stance phase but they are different during swing phase. These trajectories and angles impart the same foot-ground interaction but keep the knee fixed, while also limiting the hip motion.

### 2.3. Hip Balancing Strategies

Maintaining balance on a patient dog requires accurate stimulation of the proper muscles using position feedback from the orientation sensor on the dog. We program the dedicated Arduino micro-controller to measure the sensor, compute the corrective action using the appropriate stabilization strategy, and issue the precise control command to the muscles of the dog via electrodes. Using the bionic test-bed to evaluate the effectiveness of the closed loop balancing strategy, the stabilizing control commands are numerically added to the gait commands. This provides insight into gain adjustments and any necessary qualitative improvements.

Our three-link model in Figure 9 shows that there are many balancing strategies that can be devised and evaluated. These strategies can involve one leg or both legs, and balancing adjustments can be made during the contact or the swing phase. In this study, we present two example strategies, which use just one leg to affect body balancing. This single leg is in the stance phase of the step and the toes touch the ground. We will test the first strategy with our test-bed in a stationary standing mode where both legs touch the ground. This test result can be extended to the walking mode because the important measurement that the “balancing device” needs for body stabilization is the pelvis rotation angle, and the walking speed is not a factor.

#### 2.3.1. Strategy Number 1

As the dog walks, the open-loop gait is sufficient for some minimum level of successful walking motion. Because of the spinal injury, this motion does not receive any control feedback from the brain and eventually the pelvis of the dog begins to tilt with respect the ground and the dog starts to fall over. In our system, the IMU sensor measures this change in hip angle. Because the pelvis angle changes continuously and cyclically during the walking motion, we have used empirical observations to set a critical threshold value for the pelvis angle that indicates impending failure of the step.

**Figure 13:**
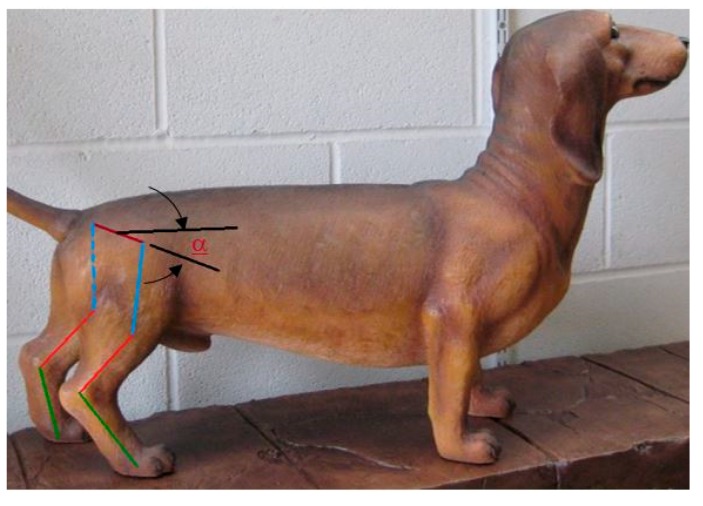
3D model of two legs. α is the hip angle with the ground.

When our sensor reads hip angles greater than the specified critical value, we can calculate the changes in leg positions that will affect a desired change in the hip position, Δx. This leg adjustment will act to level the hip and stabilize the hind quarters. Figure 13 shows the 3D model of both legs when the rear quarters of the dog is modeled using two of the two-link manipulators shown in Figure 9, one for each rear leg.

In this figure, “α” is the angle of hip rotation measured by IMU, and “h” is the width of the pelvis. When pelvis rotates, we can calculate Δx as follows:

(2) Δx=h×tan(α)

We always stimulate muscles of the leg that is in contact with the ground, either while the dog is standing or while the animal is walking. Assuming the right leg is in contact with the ground and the dog is falling toward the left side, the contact leg must cause the hip on the right to descend by some amount, Δx, in order to balance the body. However, if dog is falling toward the right side, then the contact leg must move the right side of the hip upward.

**Figure 14:**
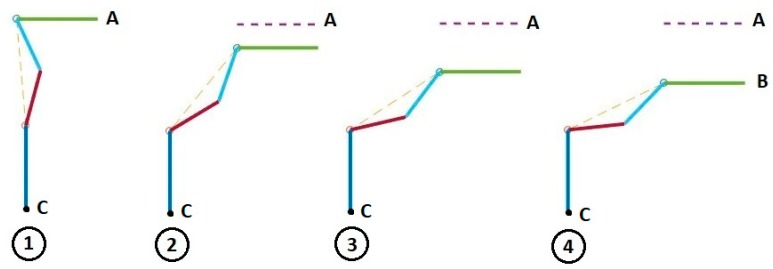
Hip position change during stimulation using strategy number 1.

**Figure 15:**
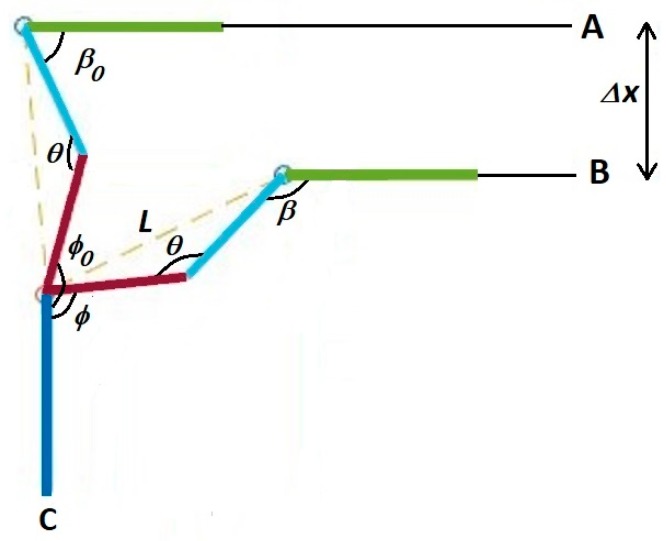
Strategy 1, raise or lower the contact leg to level the pelvis.

Figure 14 shows four positions of the contact leg in the sequence of motion necessary to move one side of the pelvis during the balancing phase. In this strategy, the muscles stimulation causes dog body to be pushed slightly forward and since the front legs of dog are healthy, the animal can adjust its center of gravity for controlling body by moving its front leg. Since forward movement of our test-bed has been constrained, the leg of robot is moved to level the hip in our tests. The geometry of the motion is shown in Figure 15. In this figure, $\beta_{0}$ is initial hip angle, β is final hip angle, $\phi_{0}$ is initial hock angle, ϕ is final hock angle. Because of our previous analysis of the knee motion during the forward gait, the knee joint, θ, is fixed. we calculate final hip and hock angles based on initial angles and the required hip motion, Δx. The effective length, L, of the tibia-femur combination is:

(3) $$L=\sqrt{L_{f}^{2}+L_{t}^{2}-2L_{f}L_{t}\cos\left(\theta \right)}$$

where $L_{f}$ and $L_{t}$ are length of femur and tibia respectively. The hip and hock angles are computed from geometry:

(4) $$\beta =\mathrm{acos}\left(\cos\left(\frac{\theta }{2}-\beta_{0}\right)-\frac{\Delta x}{L}\right)+\frac{\theta }{2}$$

(5) $$\phi =\frac{\pi }{2}+\theta -\beta$$

Since we have knowledge about the initial hip and hock angles on our bionic dog test-bed, we can program our system to calculate the final leg angles required to balance the hip and body of the dog. On the actual dog, we do not currently have these measurements, although we do have plans in the future to investigate the capability of using multiple inertial sensors on the legs in order to measure these positions.

#### 2.3.2. Strategy Number 2

**Figure 16:**
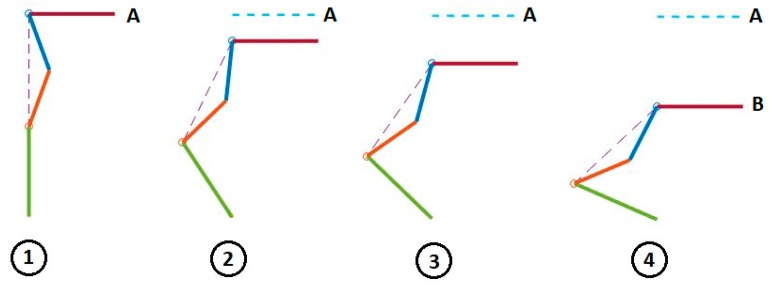
Hip position change during stimulation using strategy number 2.

The second approach differs only in that it raises or lowers the body of the dog vertically, with no forward or backward motion. This requires a change in the computation of the new hip and hock joint angles. We have shown four positions of the contact leg in this strategy in Figure 16. As in the previous strategy, the dog must adjust its body to position B to level the hip by Δx. 

**Figure 17:**
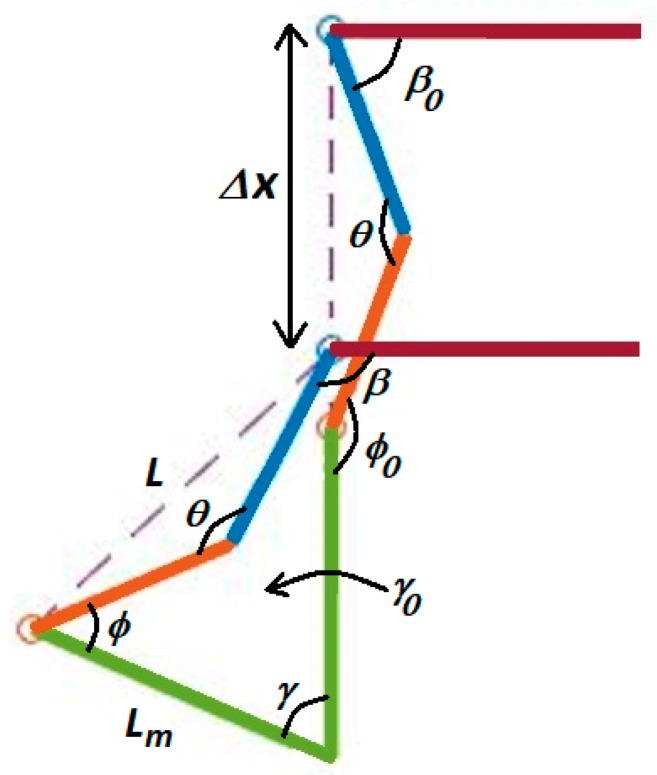
Strategy 2, move the body only in the vertical direction.

The initial position of the leg in Figure 16 has the metatarsal vertical to the ground. The sequence shows the necessary motion of the leg that will reposition the hip in order to make the hip level and balance the dog. The first and last position of the leg during balancing is shown in Figure 17.

We have introduced γ, the angle of metatarsus with the vertical. In Figure 17, the initial value of this angle, $\gamma_{0}$, as shown, is zero. It is possible that this value is not zero at initial leg position and we consider this value in our formulation. $L_{m}$ is the length of metatarsus and we can always calculate the value of $\gamma_{0}$ using our bionic dog servo position sensors:

(6) $$\gamma_{0}=a\sin\left[\frac{L}{L_{m}}\sin\left(\beta_{0}-\frac{\theta }{2}\right)\right]$$

To calculate hip angle, β, and metatarsus angle, γ, the following two equations and two unknowns can be solved:

(7) $$\{\begin{array}{l} L\cos(\beta_{0}-\frac{\theta }{2})+L_{m}\cos\left(\gamma_{0}\right)-\Delta x=L\cos(\beta -\frac{\theta }{2})+L_{m}\cos\left(\gamma \right)\\ L_{m}\sin\left(\gamma \right)-L\;\sin\left(\beta -\frac{\theta }{2}\right)=L_{m}\sin\left(\gamma_{0}\right)-L\sin\left(\beta_{0}-\frac{\theta }{2}\right) \end{array}$$

and finally, the hock joint angle, ϕ, is calculated from following equation:

(8) $$\phi =\frac{\pi }{2}-\gamma +\theta -\beta$$

**Figure 18:**
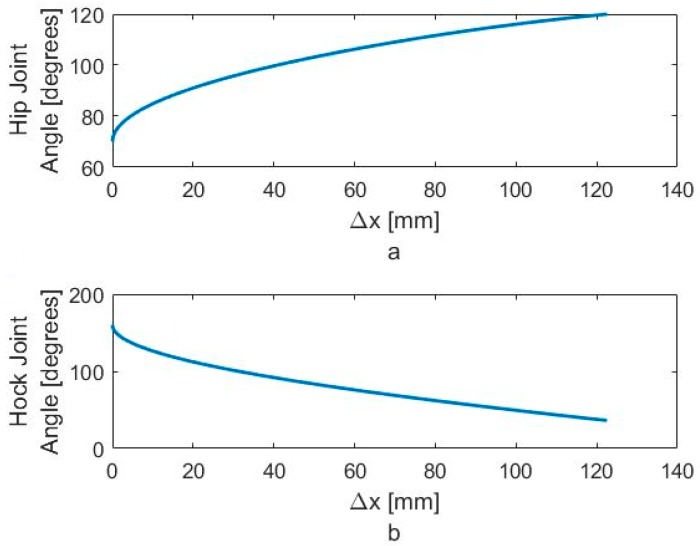
Required motion for monotonically (**a**) increasing the hip angle and (**b**) decreasing the hock angle.

In Figure 18a,b, we show the typical angle changes of the hip and hock joints during the stabilizing process. Using our robotic test bed, we can calculate the necessary joint angles to lower the hip and level the pelvis. Figure 18a shows that the required motion for monotonically increasing the hip angle and Figure 18b shows it for monotonically decreasing the hock angle. While we can do this precisely on the robotic test-bed, is neither possible nor necessary to do so on the actual dog while walking. As we sense an impending fall, we stimulate the hip and hock joints in the proper direction until the IMU indicates that the pelvis is level.

## 3. Results and Discussion

### 3.1. Experimental Results for the Bionic Dog Walking

**Figure 19:**
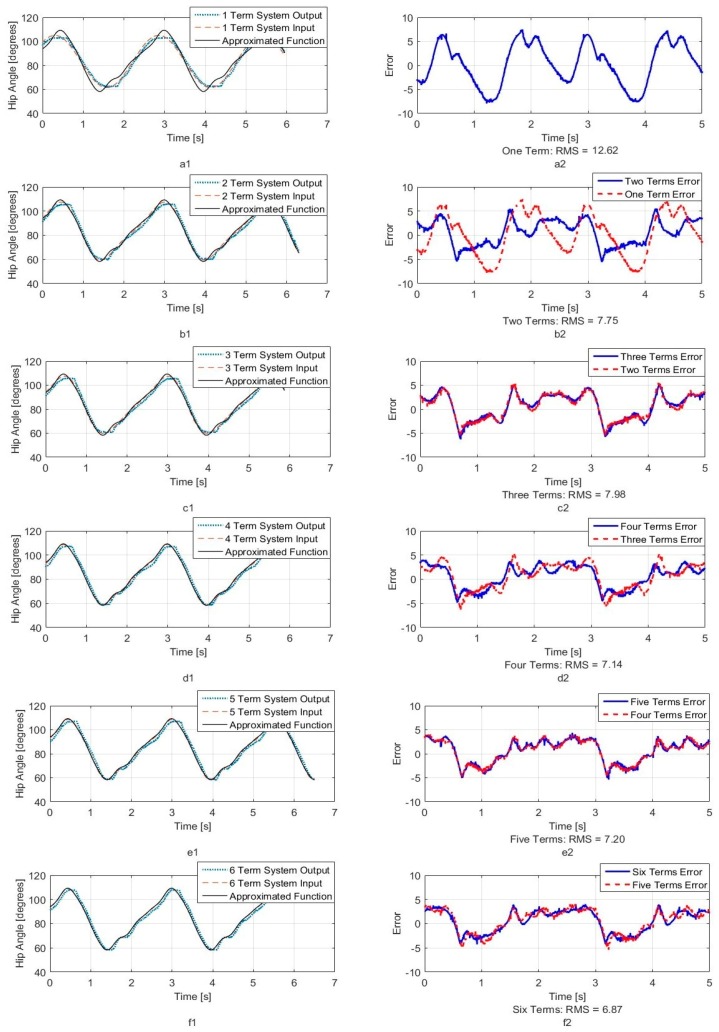
System output for hip joint angle. (**a1**) One term of Fourier series system output (**a2**) One term output error (**b1**) Two terms of Fourier series system output (**b2**) Two terms output error (**c1**) Three terms of Fourier series system output (**c2**) Three terms output error (**d1**) Four terms of Fourier series system output (**d2**) Four terms output error (**e1**) Five terms of Fourier series system output (**e2**) Five terms output error (**f1**) Six terms of Fourier series system output (**f2**) Six terms output error.

**Figure 20:**
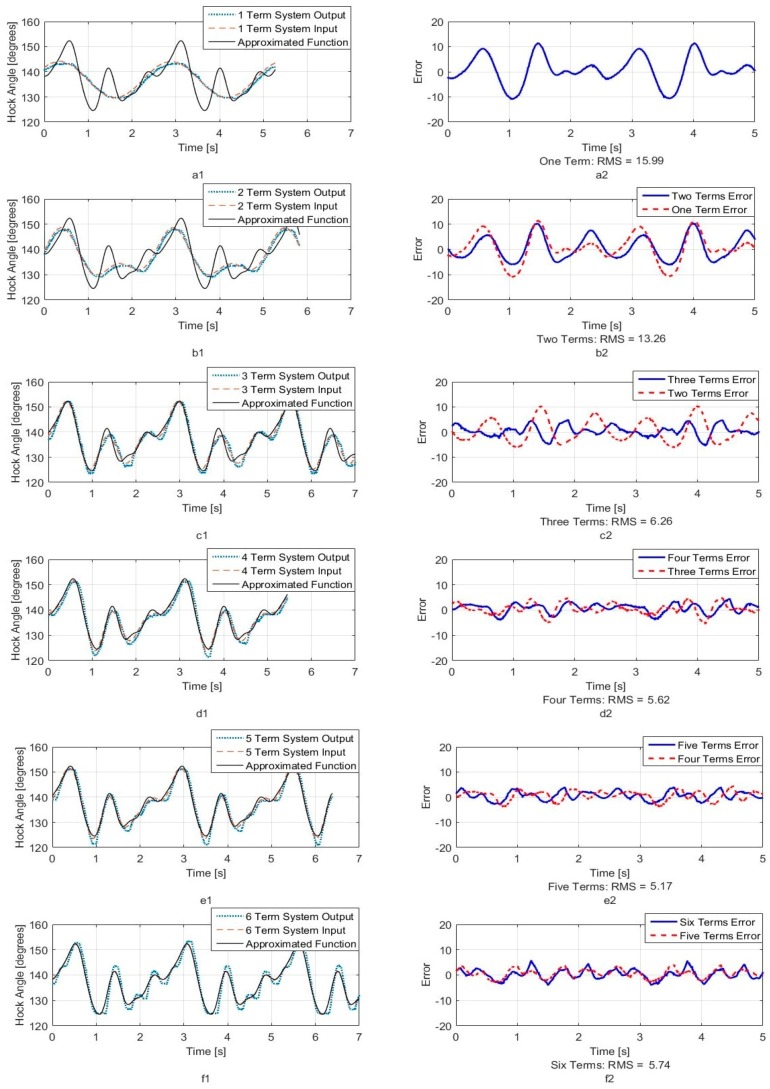
System output for hock joint angle. (**a1**) One term of Fourier series system output (**a2**) One term output error (**b1**) Two terms of Fourier series system output (**b2**) Two terms output error (**c1**) Three terms of Fourier series system output (**c2**) Three terms output error (**d1**) Four terms of Fourier series system output (**d2**) Four terms output error (**e1**) Five terms of Fourier series system output (**e2**) Five terms output error (**f1**) Six terms of Fourier series system output (**f2**) Six terms output error.

**Figure 21:**
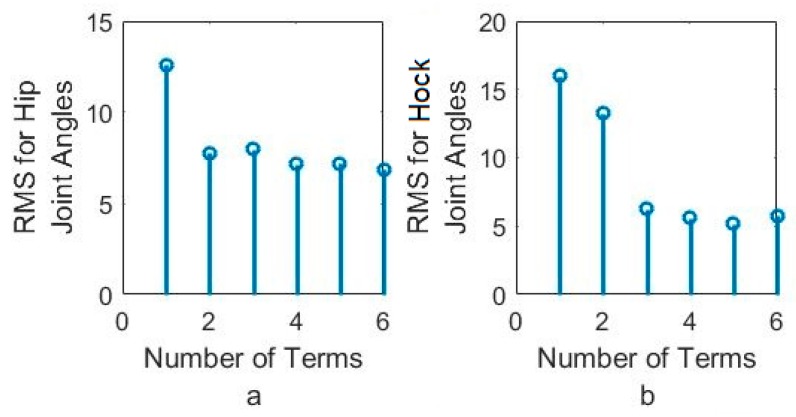
RMS error between system output and approximated function for (**a**) hip joint angles and (**b**) hock joint angles based on the number of Fourier series.

We want to match the walking gait of the bionic dog with the experimental trajectory as closely as possible. Because the swing phase does not play an important role for us, we perform our tests on the robot using the original hip and hock joint angles generated from VICON but we fix the knee joint. The two Arduino boards apply the six-term approximated functions for the hip and hock joints shown in Figure 5 and Figure 7 as the positional inputs to the joint servo motor system. We compare the experimental joint motor motion output with the six term Fourier input function. In Figure 19 and Figure 20, we look at the accuracy of the trajectory as a function of the number of Fourier sequence terms we included in joint angle approximations. Figure 19 shows the time series for the hip joint. Figure 20 shows the time series for the hock joint, along with the residual error after we add Fourier terms in the approximation. For each approximated input function, we have calculated RMS error. The RMS error plot based on number of terms for hip and hock joint angles have been shown in Figure 21a,b respectively. The experimental response shows that increasing the number of Fourier terms to higher than two for the hip and three for the hock does not significantly reduce the tracking error for these joints. These results show that we can mimic our dog gait using only three terms of Fourier series. In fact, increasing the number of Fourier terms actually increases the error, due to friction and high frequency attenuation from the mechanical components.

### 3.2. Experimental Results for Balancing Using the Bionic Dog

We assume the patient dog can stand and even walk, but the spinal cord injury prevents the dog from walking with a normal cadence. This abnormal gait may cause dog to tilt its body toward one side, and eventually falling will occur. Usually, the dog can walk for some period “open loop”, with no compensation of the real legs from the brain, but when pelvis rotation angle exceeds the critical value, the dog will fall.

Before using the device for the actual dog, we test the balancing strategy on the bionic test bed. We assume dog can walk but it is possible that the pelvis leans to one side and falling occurs. In the bionic test bed, we use the servo motors to replace the muscles. Instead of applying electrical charge to the muscles as we test the balancing strategy, the master Arduino board applies a small supplementary voltage to the motor that augments the open loop gait command. For our initial testing, we place the bionic test bed in a stationary, standing, mode. We know that the device functionality depends on the hip rotation and joint angles, and we want to validate our approach in this simpler standing configuration. For our tests, we have the bionic dog lift one leg and stand on the other, then we manually move the free leg so as to tilt the pelvis. When the pelvis reaches the critical angle, the hip and hock joints move according to our algorithm, so that the pelvis angle is stabilized.

**Figure 22:**
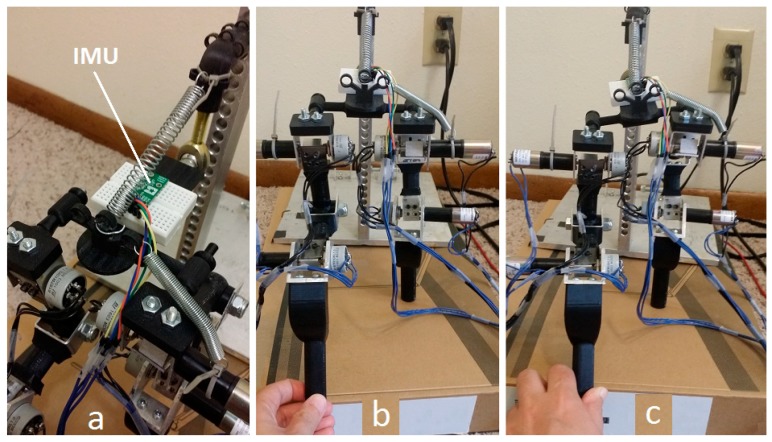
Hip balancing experiment, (**a**) IMU installed on hip, (**b**) balanced hip, (**c**) unbalanced hip.

We show this test sequence in Figure 22 where the IMU is installed on pelvis of replica (Figure 22a). When the dog is standing on one leg with the hip level, the IMU reads close to zero (Figure 22b). As we move the free leg, we induce and angle on the pelvis (Figure 22c).

**Figure 23:**
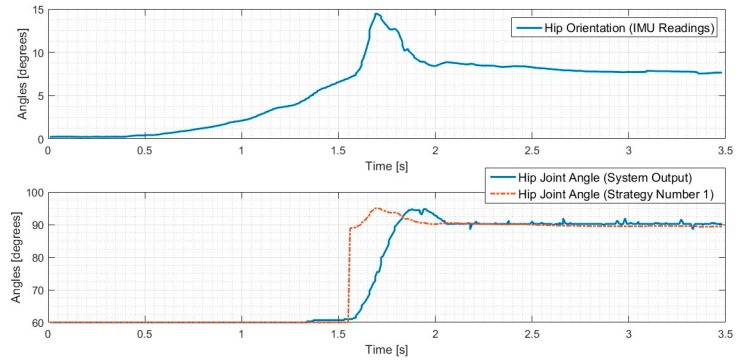
Hip balancing test results for hip joint angle changes.

**Figure 24:**
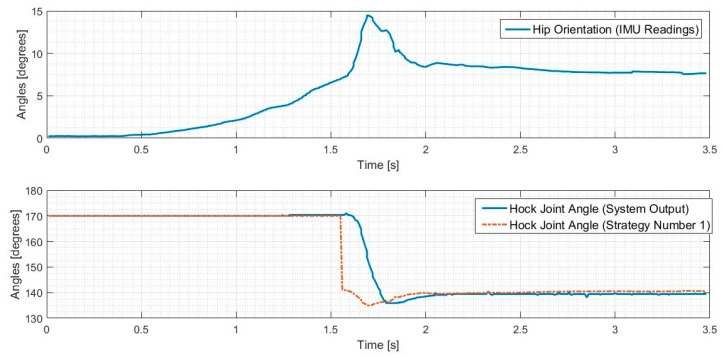
Hip balancing test results for hock joint angle changes.

The results of this stationary test are shown in Figure 23 and Figure 24, where the IMU initially shows that the pelvis is level. As the IMU reads the increasing pelvis angle, eventually the pelvis exceeds the critical value of 10 degrees and initiates the stabilization response. The microcontroller calculates desired hip and hock joint angles based on the excessive pelvis tilt, and applies corrective commands to both the hip and hock joints. The stabilization commands monotonically decrease the pelvis orientation angle and then stop when the IMU reads an angle within the acceptable range.

**Figure 25:**
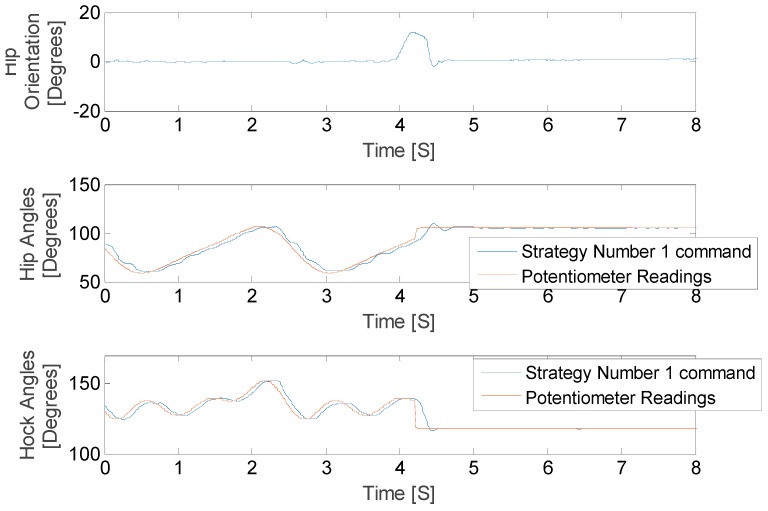
Hip balancing test results for hip joint angle changes.

**Figure 26:**
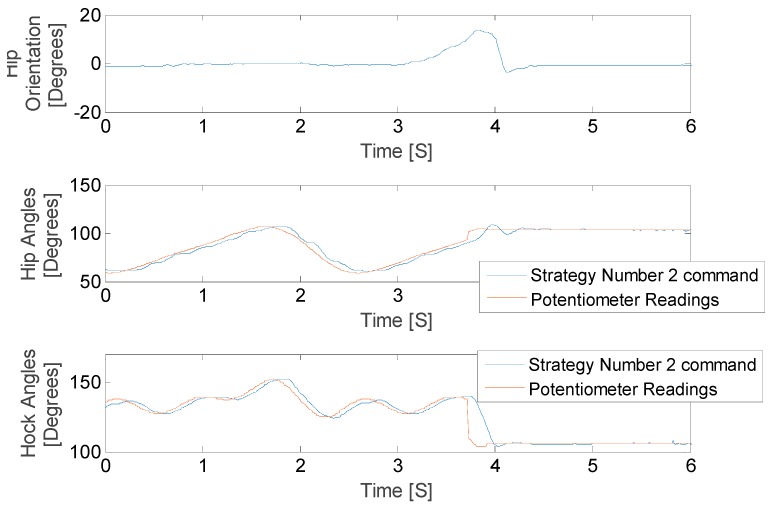
Hip balancing test results for hock joint angle changes.

We have defined the open loop gait that we will use to drive the leg motion during walking. Once we validated the algorithms in the stationary mode, we ran the robot dog with three Fourier term approximation input and perturbed it such that the pelvis exceeded the critical value. The results of these tests for both balancing strategies are shown in Figure 25 and Figure 26, where the IMU initially shows that the pelvis is level. As the IMU reads the increasing pelvis angle, eventually the critical value of 10 degrees is exceeded, and the stabilization response is initiated. The microcontroller calculates desired hip and hock joint angles based on the excessive pelvis tilt, and applies corrective commands to both the hip and hock joints. The stabilization commands monotonically decrease the pelvis orientation angle and then stops when the IMU reads an angle within the acceptable range.

As shown in Figure 25 and Figure 26 the balancing tactic does not depend on the history of the leg motion, but rather only on the pelvis orientation, the hip and hock joints angles just at the time of excessive pelvis angle, and the balancing strategy that is selected. The difference between the two strategies is the final posture of the leg and body after stimulation. We stop the test in both cases once the pelvis is level. While both approaches are successful, each strategy depends on the specific motion trajectory and may hold value associated with the unique dog injury and reflexive capabilities.

## 4. Conclusions

In this study, we designed and built a “balancing device” and a bionic test-bed to test the stabilization methods for rear quarters of dogs with spinal cord injuries using FES. We showed a compact and efficient Arduino microcontroller can be used as the core of our balancing device. We showed this platform can acquire the data from IMU sensor, compute the stimulation command, and it is capable of generating pulsed voltage commands for stimulation. Additional electronic hardware is necessary to generate the required voltages, but this equipment is available in a small, compact footprint as well.

We modeled the dog skeleton as a three-link manipulator and studied the typical dog gait. Using information that we obtained from VICON and our MATLAB (MathWorks, Natick, MA, USA) simulations, we mimicked the gait using our test-bed. This gait study helped assure us that our test-bed is a proper mechanism to mimic dog legs movements, and thus useful for balancing algorithm development. We present results for two algorithmic approaches when the bionic dog was both standing and walking. Results of our experiments show the device can sense the critical orientation and can apply a corrective stimulation command to achieve balance. We have this hope that the device can help restore normal walking to patient dogs. The device can be programmed properly for each individual patient based on their abnormal walking gait.

Although the balancing device can control signals to the leg motors of the bionic replica, the next step is to investigate the stimulation and response of actual dog muscles during walking. This will require developing the electrical stimulation hardware and mapping the electrode placement to specific leg response.

## Figures and Tables

**Table 1:** Fourier series constants for approximated functions.

Constant	Hip Joint	Knee Joint	Hock Joint
***a*_0_**	83.45	137.3	137.1
***a*_1_**	15.95	6.07	4.723
***b*_1_**	14.84	−6.017	5.428
***a*_2_**	−5.337	−1.56	−1.919
***b*_2_**	0.6666	7.131	4.089
***a*_3_**	0.0105	−0.9911	−1.379
***b*_3_**	−0.4825	−3.915	−5.815
***a*_4_**	−0.9902	−1.32	0.5557
***b*_4_**	−0.9245	0.4471	1.19
***a*_5_**	0.1773	0.1738	0.3494
***b*_5_**	0.2179	0.3888	−0.8079
***a*_6_**	0.5627	0.1626	−1.126
***b*_6_**	−0.2534	0.09822	0.2147
